# Prognostic value of combination of preoperative platelet count and mean platelet volume in patients with resectable non-small cell lung cancer

**DOI:** 10.18632/oncotarget.14921

**Published:** 2017-01-31

**Authors:** Liuwei Gao, Hua Zhang, Bin Zhang, Lianmin Zhang, Changli Wang

**Affiliations:** ^1^ Department of Lung Cancer, Tianjin Medical University Cancer Institute and Hospital, National Clinical Research Center for Cancer, Tianjin, China; ^2^ Department of Lung Cancer, Tianjin Medical University Cancer Institute and Hospital, Key Laboratory of Cancer Prevention and Therapy, Tianjin, China; ^3^ Department of Lung Cancer, Tianjin Medical University Cancer Institute and Hospital, Tianjin Lung Cancer Center, Tianjin, China

**Keywords:** combination of platelet count and mean platelet volume, non-small cell lung cancer, thrombocytosis, mean platelet volume, prognosis

## Abstract

The aim of the present study was to investigate the prognostic value of the combination of preoperative platelet count (PLT) and mean platelet volume (MPV) in patients with primary operable non-small cell lung cancer (NSCLC). We retrospectively analysed data from 546 patients with NSCLC who underwent complete resection at our institution from 2006 to 2010. Patients’ clinical characteristics and laboratory test data at initial diagnosis were collected. Both preoperative PLT and MPV (COP-MPV) were calculated on the basis of the data obtained using the recommended cut-off values of 300 × 10^9^ L^−1^ and 11.0 fL, respectively. Patients with both an elevated PLT (≥300× 10^9^ L^−1^) and a decreased MPV (<11.0 fL) were assigned a score of 2, and patients showing one or neither were allocated a score of 1 or 0, respectively. Multivariate analysis of the 9 clinical laboratory variables selected by univariate analysis revealed that preoperative COP-MPV was a significantly independent prognostic factor for overall survival (OS) (hazard ratio, 1.775; 95% confidence interval, 1.500–2.101; *P*< 0.001) and disease-free survival (DFS) (hazard ratio, 1.719; 95% confidence interval, 1.454–2.033; *P*< 0.001). In subgroup analyses for tumour pathological stage (I/II/IIIA) patients, we found that the level of COP-MPV was significantly associated with OS and DFS in each subgroup (*P*< 0.001, *P*< 0.001, *P*<0.001 for OS and *P*<0.001, *P*< 0.001, *P*=0.001 for DFS, respectively). In conclusion, the preoperative COP-MPV is a promising predictor of postoperative survival in patients with NSCLC and could classify these patients into three independent groups before surgery.

## INTRODUCTION

Non-small cell lung cancer (NSCLC), which accounts for approximately 80% of all lung cancers, is the most common cause of cancer-related deaths worldwide [1]. Despite recent improvements in chemoradiation therapy, radical resection and targeted therapies, the clinical prognosis of lung cancer remains poor, with a 5-year survival rate of less than 15% [2]. Recently, several publications have reported prognostic predictors for patients with NSCLC; however, most of these survival-related factors cannot be obtained preoperatively. Moreover, some of these factors are only used as research tools.

In the last decade, several studies have revealed that platelet activation is an important biological process in metastasis and carcinogenesis [3–6]. Recently, the relation between thrombocytosis and poor prognosis of multiple solid tumours, such as lung, gastric, ovarian and colon cancers [7–10], has been reported. The mean platelet volume (MPV), which is a platelet volume index [11], is considered to be a hallmark of platelet activation and is generally used for routine analysis during complete blood counts [12]. Recent studies have revealed that MPV levels are abnormal in patients with various disorders, including malignant tumours [13–18]. In addition, a recent study has investigated the prognostic value of combining the PLT with MPV (COP-MPV) to predict post-surgical survival in oesophageal squamous cell cancer (ESCC) patients [19]. However, studies regarding the prognostic impact of PLT combined with MPV in patients with resected NSCLC have not been reported. In this study, therefore, we retrospectively evaluated the prognostic significance of preoperative COP-MPV in primary operable patients with NSCLC.

## RESULTS

A total of 546 NSCLC patients satisfying the inclusion criteria were enrolled in this study, including 119 patients with COP-MPV = 0; 295 patients with COP-MPV = 1; and 132 patients with COP-MPV = 2. Among these patients, 183 (33.5%) were women, and 363 (66.5%) were men. The median age of the participants was 60 years (range: 24–82 years). The pathological stages were distributed as follows: 236 patients with stage I, 113 patients with stage II and 197 patients with stage IIIA. The median follow-up time was 44.6 (2–96) months. In our study, 303 patients died during the observation period.

Table 1 shows the relationship between COP-MPV and the clinical characteristics of the studied patients with NSCLC. No significant differences were found among the three groups when divided by COP-MPV, except for sex (*P*=0.004) and pathological stage (I/II/IIIA) (*P*<0.001).

**Table 1:** Association of COP-MPV with the clinicopathologic characteristics of patients with NSCLC

Variables	COP-MPV = 0 n(%)	COP-MPV = 1 n(%)	COP-MPV = 2 n(%)	*P* value
**Age (year)**				0.890
** ≤60**	62(52.1)	146(49.5)	66(50.0)	
** >60**	57(47.9)	149(50.5)	66(50.0)	
**Sex**				0.004
** Female**	53(44.5)	82(27.8)	48(34.1)	
** Male**	66(55.5)	213(72.2)	84(65.9)	
**Smoking**				0.161
** Yes**	70(58.8)	202(68.5)	89(67.4)	
** No**	49(41.2)	93(31.5)	43(32.6)	
**Tumor location**				0.385
** Right**	67(56.3)	173(58.6)	85(64.4)	
** Left**	52(43.7)	122(41.4.)	47(35.6)	
**Histological subtype**				0.135
** Adenocarcinoma**	58(48.7)	119(40.3)	43(32.6)	
** SqCC**	46(38.7)	137(46.4)	70(53.0)	
** Others**	15(12.6)	39(13.3)	19(14.4)	
**Lesion**				0.411
** Peripheral**	91(76.5)	213(72.2)	91(68.9)	
** Central**	28(23.5)	82(27.8)	41(31.1)	
**Resection type**				0.860
** Pneumonectomy**	14(11.8)	35(11.9)	18(13.6)	
** Lobectomy**	105(88.2)	260(88.1)	114(86.4)	
**Lymph node metastasis**				0.810
** Yes**	51(42.9)	136(46.1)	58(43.9)	
** No**	68(57.1)	159(53.9)	74(56.1)	
**Pathological stage**				<0.001
** I**	55(46.2)	141(47.8)	40(30.3)	
** II**	32(26.9)	32(10.8)	49(37.1)	
** IIIA**	32(26.9)	122(41.4)	43(32.6)	

Abbreviations: COP-MPV, combination of preoperative platelet count and mean platelet volume; NSCLC, non-small cell lung cancer; SqCC, Squamous cell carcinoma.

*P* less than .05 is statistically significant.

Table 2 shows the distribution of clinicolaboratory characteristics in the three groups divided according to the COP-MPV. There were significant differences among the three groups in haemoglobin (Hb) (*P*<0.001), alkaline phosphatase (ALP) (*P*=0.019), WBC count (*P*<0.001), platelet count (*P*<0.001), MPV (*P*<0.001), red blood cell distribution width (RDW) (*P*=0.019), albumin (*P*=0.004), D-dimer (*P*=0.049), fibrinogen (*P*<0.001), maximum tumour diameter (*P*<0.001) and survival period (*P*<0.001).

**Table 2:** Association of COP-MPV with the clinicolaboratory characteristics of patients with NSCLC

Variables	COP-MPV=0(n=119)	COP-MPV=1(n=295)	COP-MPV=2(n=132)	*P* value
**Age (year)**	59.8±8.4	60.9±9.7	59.9±9.2	0.423
**Hb (gL^-1^)**	139.2±14.5	141.1±14.0	134.7±19.7	<0.001
**LDH (UL^-1^)**	178.4±36.8	185.7±60.9	180.6±45.1	0.961
**ALP (UL^-1^)**	74.2±26.6	72.5±19.8	81.8±33.6	0.019
**WBC count(×10^3^/μL)**	6.4±1.8	6.8±1.5	7.4±1.8	<0.001
**PLT (×10^9^L^-1^)**	231.2±25.7	261.7±46.7	343.7±65.2	<0.001
**MPV (fL)**	12.0±1.3	10.8±0.3	10.6±0.4	<0.001
**RDW (%)**	13.2±0.9	13.0±0.8	13.4±1.6	0.019
**Albumin (gL^-1^)**	43.4±3.9	43.1±4.1	41.8±4.5	0.004
**D-dimer (mgL^-1^)**	0.18±0.11	0.21±0.28	0.20±0.12	0.049
**Fibrinogen (gL^-1^)**	3.5±0.9	3.5±0.9	4.2±1.0	<0.001
**Maximum tumor diameter (cm)**	3.9±1.9	4.1±1.9	5.4±2.5	<0.001
**Survival period (m)**	57.0±19.6	42.6±25.4	37.9±24.5	<0.001

Abbreviations: COP-MPV, combination of preoperative platelet count and mean platelet volume; NSCLC, non-small cell lung cancer; Hb, hemoglobin; LDH, lactate dehydrogenase; ALP, alkaline phosphatase; WBC, white blood cell; PLT, platelet count; MPV, mean platelet volume; RDW, red blood cell distribution width.

*P* less than .05 is statistically significant.

Based on the cut-off values, we divided the patients into different groups. Analyses of overall survival (OS) in relation to platelet count and MPV were carried out. Univariate analyses showed that platelet count and MPV were significant factors, with a hazard ratio (HR) =1.718 (95% confidence interval [CI], 1.348–2.191) for platelet count and a HR =2.220 (95% CI, 1.585–3.108) for MPV. To determine the optimal factor for patient prognosis, we assessed the prognostic value of COP-MPV. Specifically, the indicator (COP-MPV) was found to have high prognostic value, with a HR of 1.859 (95% CI, 1.574–2.196). For analyses of disease-free survival (DFS), the HR for COP-MPV was 1.841 (95% CI, 1.558–2.174) (Table 3). Other factors, including lesion, pathological stage, resection type, RDW, albumin, D-dimer, ALP and fibrinogen were also considered to be significant for DFS and OS.

**Table 3:** Univariate analysis for disease-free survival and overall survival

Variables	*P* value	DFSHR (95 % CI)	*P* value	OSHR (95 % CI)
**Age (≤60vs >60)**	0.885	1.017(0.812–1.274)	0.824	1.026(0.819 –1.285)
**Sex (male vs female)**	0.647	0.945(0.742 – 1.203)	0.366	0.895(0.703 – 1.139)
**Lesion (peripheral vs central)**	0.028	1.313(1.030 –1.674)	0.020	1.336(1.048 – 1.703)
**Pathological stage (I, II, IIIA)**	<0.001	1.848(1.618– 2.111)	<0.001	1.846(1.616 – 2.110)
**Smoking (yes vs no)**	0.822	0.973(0.766 – 1.236)	0.387	0.900(0.708– 1.143)
**Tumor location (left vs right)**	0.630	0.945(0.752 –1.188)	0.619	0.944(0.751– 1.186)
**Histological subtype (adenocarcinoma, SqCC, others)**	1.000	1.000(0.849 –1.178)	0.965	0.996(0.845– 1.174)
**Resection type (pneumonectomy, lobectomy)**	0.032	1.414(1.030 – 1.940)	0.044	1.385(1.009 – 1.902)
**RDW (≥13.0 vs <13.0 %)**	0.020	1.310(1.044 – 1.642)	0.016	1.319(1.052 – 1.654)
**Hb (≥120 vs <120 gL^-1^)**	0.058	0.711(0.500 – 1.011)	0.070	0.722(0.507 – 1.027)
**Albumin (>40 vs ≤40 gL^-1^)**	0.021	1.340(1.045 – 1.718)	0.044	1.292(1.007 – 1.658)
**WBC count(≥6.685vs <6.685×10^3^/μL)**	0.075	1.228(0.979 – 1.538)	0.052	1.251(0.998 – 1.568)
**PLT (≥300 vs <300 ×10^9^L^-1^)**	<0.001	1.698(1.332– 2.166)	<0.001	1.718(1.348 – 2.191)
**MPV (≥11.0vs <11.0 fL)**	<0.001	2.176(1.554– 3.046)	<0.001	2.220(1.585– 3.108)
**D-dimer (>0.1vs ≤0.1 mgL^-1^)**	0.007	1.367(1.090–1.715)	0.045	1.262(1.005– 1.584)
**LDH (≥174vs <174UL^-1^)**	0.205	1.157(0.923 – 1.450)	0.204	1.158(0.924– 1.451)
**ALP (≥71vs <71UL^-1^)**	0.031	1.284(1.023 – 1.611)	0.039	1.269(1.012 – 1.592)
**Fibrinogen (≥3.543vs <3.543gL^-1^)**	<0.001	1.059(1.241 – 1.960)	<0.001	1.580(1.257 – 1.985)
**COP-MPV (0,1,2)**	<0.001	1.841(1.558 – 2.174)	<0.001	1.859(1.574 – 2.196)

Abbreviations: DFS, disease-free survival; OS, overall survival; HR, hazard ratio; CI, confidence interval; SqCC, Squamous cell carcinoma; RDW, red blood cell distribution width; Hb, hemoglobin; WBC, white blood cell; PLT, platelet count; MPV, mean platelet volume; LDH, lactate dehydrogenase; ALP, alkaline phosphatase; COP-MPV, combination of preoperative platelet count and mean platelet volume.

*P* less than .05 is statistically significant.

Multivariate analyses using the 9 significant variables above (excluding platelet count and MPV) were performed with the Cox proportional hazards model. In that model, we demonstrated that preoperative COP-MPV was significantly related to DFS and OS (HR, 1.719; 95%CI, 1.454–2.033; *P*<0.001 and HR, 1.775; 95%CI, 1.500–2.101; *P*<0.001, respectively) along with pathological stage and RDW levels (Table 4).

**Table 4:** Multivariate analysis for disease-free survival and overall survival

Variables	*P* value	DFSHR (95 % CI)	*P* value	OSHR (95 % CI)
**Lesion (peripheral vs central)**	0.427	1.114(0.854 – 1.452)	0.390	1.125(0.861 – 1.470)
**Pathological stage (I, II, IIIA)**	<0.001	1.794(1.563 –2.059)	<0.001	1.830(1.594– 2.101)
**Resection type (pneumonectomy, lobectomy)**	0.435	1.147(0.813 – 1.620)	0.592	1.100(0.777– 1.556)
**RDW (≥13.0 vs <13.0 %)**	0.019	1.317(1.046 – 1.657)	0.009	1.360(1.081– 1.712)
**Albumin (>40 vs ≤40 gL^-1^)**	0.913	1.015(0.782 – 1.316)	0.899	0.983(0.757 – 1.277)
**D-dimer (>0.1vs ≤0.1 mgL^-1^)**	0.371	1.114(0.880– 1.410)	0.720	1.044(0.824– 1.322)
**ALP (≥71vs <71UL^-1^)**	0.747	1.040(0.820– 1.319)	0.857	1.022(0.806 – 1.296)
**Fibrinogen (≥3.543vs <3.543gL^-1^)**	0.225	1.164(0.910– 1.490)	0.114	1.217(0.954– 1.554)
**COP-MPV (0,1,2)**	<0.001	1.719(1.454– 2.033)	<0.001	1.775(1.500 – 2.101)

Abbreviations: DFS, disease-free survival; OS, overall survival; HR, hazard ratio; CI, confidence interval; RDW, red blood cell distribution width; ALP, alkaline phosphatase; COP-MPV, combination of preoperative platelet count and mean platelet volume.

*P* less than .05 is statistically significant.

**Figure 1:**
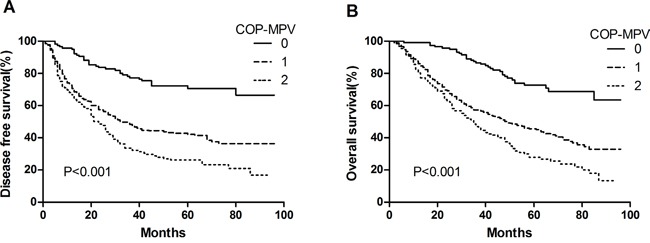
**A**. Kaplan–Meier curve for DFS of 546 NSCLC patients stratified by COP-MPV. **B**. Kaplan–Meier curve for OS of 546 NSCLC patients stratified by COP-MPV.

**Figure 2:**
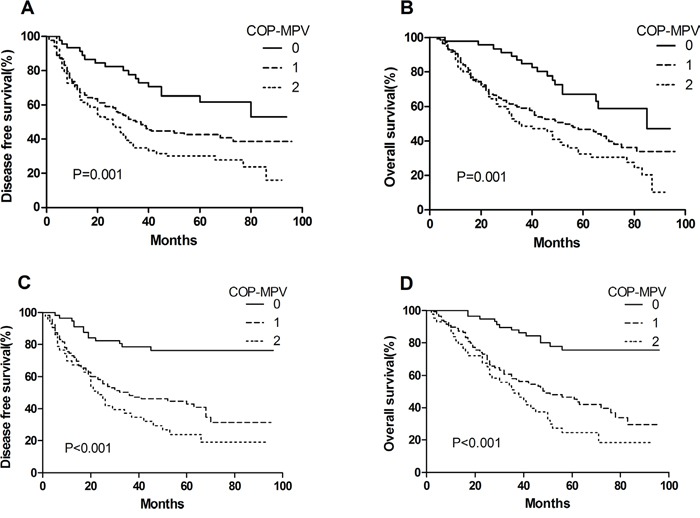
**A**. Kaplan–Meier curve for DFS of patients with SqCC stratified by COP-MPV. **B**. Kaplan–Meier curve for OS of patients with SqCC stratified by COP-MPV. **C**. Kaplan–Meier curve for DFS of patients with adenocarcinoma stratified by COP-MPV. **D**. Kaplan–Meier curve for OS of patients with adenocarcinoma stratified by COP-MPV.

**Figure 3:**
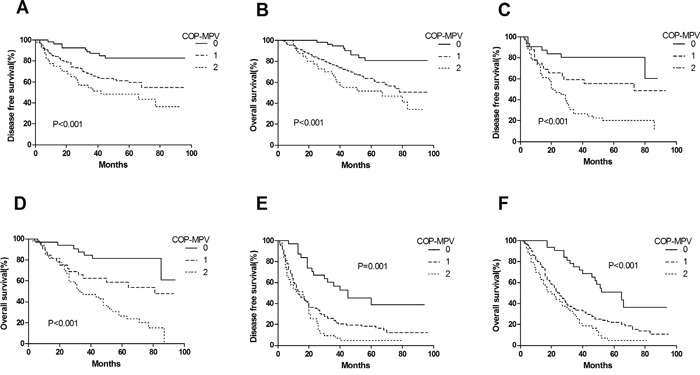
**A**. Kaplan–Meier curve for DFS of pathological stage I NSCLC patients stratified by COP-MPV. **B**. Kaplan–Meier curve for OS of pathological stage I NSCLC patients stratified by COP-MPV. **C**. Kaplan–Meier curve for DFS of pathological stage II NSCLC patients stratified by COP-MPV. **D**. Kaplan–Meier curve for OS of pathological stage II NSCLC patients stratified by COP-MPV. **E**. Kaplan–Meier curve for DFS of pathological stage IIIA NSCLC patients stratified by COP-MPV. **F**. Kaplan–Meier curve for OS of pathological stage IIIA NSCLC patients stratified by COP-MPV.

By Kaplan-Meier analysis and log-rank test, significant differences in DFS and OS among the three COP-MPV groups were demonstrated (*P*<0.001 and *P*<0.001, respectively) (Figures 1A and 1B). Patients with COP-MPV = 2 had worse prognoses than those with COP-MPV = 0 or COP-MPV = 1. The 5-year survival rate for groups 0, 1, and 2 were 72.7%, 44.4% and 27.9%, respectively. Therefore, we could clearly classify the patients into three independent groups using the preoperative COP-MPV. In the same way, COP-MPV was also able to divide the patients into three independent groups in adenocarcinoma or SqCC (Figure 2A, 2B, 2C and 2D). Furthermore, in subgroup analyses, COP-MPV showed predictive value in patients with pathological stage I, II, and IIIA tumours, whereas the lower COP-MPV group tended to have a better prognosis than the higher COP-MPV group (Figures 3A, 3B, 3C, 3D, 3E and 3F).

## DISCUSSION

To date, approximately 10% to 57% of patients with solid tumours have been shown to have thrombocytosis [20]. Recently, several studies have shown that a high PLT indicated poor postoperative survival associated with several different solid tumours [8, 10, 21, 22]. MPV was considered to be a hallmark of platelet activation [12] and indicated the prognosis in patients with malignant tumours, although the relationship between MPV and overall survival remains controversial [17, 18, 23]. Furthermore, the prognostic value of COP-MPV for post-surgical survival in ESCC has been demonstrated [19]. Hence, it is reasonable to combine PLT and MPV into a prognostic scoring system to measure platelet activation and assess the prognosis of patients with cancer. We recruited 546 NSCLC patients undergoing complete resection to evaluate the prognostic value of COP-MPV and confirmed that preoperative COP-MPV is an independent prognostic factor in this cohort.

In this study, our prominent value for platelet count based on ROC curves was 291 × 10^9^ L^−1^. It is well known that a cut-off value for reactive thrombocytosis has not been defined, although the normal high value for platelet count is 300 × 10^9^ L^−1^. Based on our ROC curve, we used 300 × 10^9^ L^−1^ as the cut-off value for platelet count. To date, a number of possible mechanisms have been proposed for why PLT is associated with poor prognosis of cancer patients. First, increased PLT may promote the growth and invasion of tumour cells. Plasma components stored in platelets contribute to cancer progression by releasing cytokines and growth factors such as VEGF, platelet-derived growth factor (PDGF), TGF-β and FGF [24, 25], which play important roles in regulating angiogenesis, cell survival and cell motility [20, 26]. In addition, platelets facilitate the generation of capillary-like structures by endothelial cells through integrins mediating cell-cell adhesion [27]. Second, by interacting with fibrin and tumour cells, platelets can promote the formation of platelet-fibrin-tumour cells, which plays a significant role in evading immune surveillance and enhancing tumour cell survival [28]. Based on the above results, previous studies have investigated the effect of platelet counts on the prognostic value in NSCLC patients and shown that platelet count could serve as a prognostic factor [7, 21].

MPV, one of the indexes marking platelet volume, is considered to be an effective marker of platelet activation. Assessment of the MPV in patients with malignant tumours has recently attracted substantial interest even though some results have been controversial. For instance, previous studies have reported that the MPV was higher in patients with gastric cancer than in control patients and that the MPV/PC (platelet count) ratio was meaningfully increased in patients with hepatocellular carcinoma [29, 30]. In addition, published research has reported a significant correlation between high MPV and advanced cancer, such as gastric cancer [16], hepatocellular carcinoma [30], endometrial cancer [15], breast cancer [31], and colon cancer [32]. It has been proposed that inflammatory cytokines, such as interleukin-6, produced by tumour cells may stimulate the differentiation and proliferation of megakaryocytes to produce abundant giant platelets. However, opposite views have also been reported. A study concerning the MPV in patients diagnosed with different cancers found a significant reduction in MPV values between patients at the time of diagnosis of a thrombotic event and control groups at the time of diagnosis [33]. Additionally, several studies have found the MPV value to be significantly lower in cancer patients with lung cancer or metastasis to the bone marrow compared to control groups [23, 34]. Furthermore, two recent studies showed that low MPV levels correlated with unfavourable prognosis in NSCLC [17, 18]. The low MPV value found in these studies might have been caused by the tendency of larger platelets to respond to stimuli compared to smaller platelets, which may lead to selective consumption of these platelets [17, 18]. Based on the above observations, our study in patients with NSCLC supported the latter views. In this study, based on ROC curve analysis, we confirmed that the ideal cut-off value of MPV was 11.0 for predicting survival in patients following complete resection of stage I-IIIA NSCLC; however, the cut-off values were different in other studies [15–19].

Hence, using platelet count and MPV for COP-MPV has good prognostic value for patients with cancer. We performed a multivariate analysis using multiple clinicolaboratory variables and found that COP-MPV correlated with DFS and OS, as well as pathological stage and RDW level. Based on Kaplan-Meier curves and log-rank tests, we determined that the preoperative COP-MPV could separate the patients into three independent groups. Moreover, when we investigated the predictive utility of the COP-MPV in patients with pathological stage I, II or IIIA separately, a significant relationship was found between the COP-MPV score and both DFS and OS for each pathological stage. Finally, our study also showed that there was a significant association between the COP-MPV level and both DFS and OS in patients with SqCC or adenocarcinoma. Preoperative peripheral blood sampling is routinely performed for NSCLC patients undergoing complete resections, making it easy to calculate both the platelet count and MPV. In comparison with serum tumour markers, hematologic markers are less costly and therefore are likely be used more extensively.

In conclusion, the preoperative hematologic marker COP-MPV can be used as an independent prognostic marker for patients with NSCLC and can classify these patients into three independent groups before surgery. To the best of our knowledge, this is the first study to assess the effect of COP-MPV on the prognosis of NSCLC patients undergoing complete resection. Large-scale prospective studies will need to be performed to validate these preliminary results.

## MATERIALS AND METHODS

### Patients

We conducted a retrospective study of patients with histopathologically confirmed NSCLC who underwent complete pulmonary resection and systematic node dissection of the hilar and mediastinal lymph nodes at the Tianjin Medical University Cancer Institute and Hospital from January 2006 to December 2010. Patients were excluded from the study if they had any of the following characteristics: (1) preoperative treatment, such as radiotherapy or chemotherapy; (2) minimal residual tumour; (3) advanced disease including malignant pleural effusion/involvement or distant metastasis; (4) evidence of infection, hematological or concomitant autoimmune diseases; (5) history of lung cancer, transplantation or immunosuppression. Based on the exclusion criteria, 546 NSCLC patients were included in the present study. All patients underwent preoperative evaluation, including clinical history, physical examination, biochemical tests, coagulation status, and complete blood cell counts. Further examination included flexible bronchoscopy, pulmonary function tests, radionuclide bone scans, and radiographic imaging (computed tomography (CT) or magnetic resonance imaging (MRI)). Tumour stages were evaluated according to the 7th edition of the TNM classification [35]. The histological classification of NSCLC was based on the WHO guidelines [36]. This retrospective study was approved by the Ethical Committees of Tianjin Medical University Cancer Institute and Hospital.

### Definition of COP-MPV

Preoperative peripheral venous blood sampling was collected from each patient for a routine laboratory full blood test one week prior to surgery. Receiver operating characteristic (ROC) curve analysis was used to determine the cut-off values for the preoperative platelet count and MPV. In our study, an MPV of 11.0 fL yielded maximum combined sensitivity and specificity on the ROC curve. The area under the curve for MPV was 0.611. Therefore, the recommended cut-off value for MPV was 11.0. Similarly, the optimal point on the ROC curve indicated a cut-off value of 291 × 10^9^ L^−1^ for the platelet count and the area under the ROC curve was 0.615. Thus, we used 300 × 10^9^ L^−1^ as the recommended cut-off value for preoperative platelet count. The COP-MPV score was calculated on the basis of data obtained as follows: patients with both an elevated PLT (≥300 × 10^9^ L^−1^) and a decreased MPV (<11.0 fL) were assigned a score of 2, and patients showing one or neither were allocated a score of 1 or 0, respectively.

### Statistical analysis

Statistical analyses were conducted using SPSS 16.0 software (SPSS Inc., Chicago, USA). Overall survival (OS) was calculated from the date of surgery to the date of patients’ death for any reason or the time the patient was last known to be alive. Disease-free survival (DFS) was defined as the time from operation to the time of first recurrence. Continuous variables are listed as the mean ± s.d, while all categorical variables are presented as frequency (percentage). Kruskal-Wallis and chi-square tests were used to analyse the differences among the groups. The prognostic analysis was performed by using univariate and multivariate Cox regression models.

Univariate analysis was used to determine which clinicolaboratory variables, including age, sex, smoking status, tumour location, lesion, pathological stage, resection type, histological subtype, RDW, WBC count, PLT, MPV, albumin, Hb, lactate dehydrogenase (LDH), ALP, fibrinogen, D-dimer and COP-MPV, were able to predict DFS and OS. Specifically, the cut-off values of clinicolaboratory variables defined by using ROC curve analyses were used to divide the patients into two groups. Clinicolaboratory variables selected by univariate analysis with a *P* value<0.05 were brought into the multivariate analysis to evaluate their independency. The survival differences among the three COP-MPV groups were determined using Kaplan-Meier analysis and the log-rank test. A *P* value less than 0.05 was considered statistically significant.
